# *Paragonimus proliferus* Metacercaria-derived antigens mitigate DSS-Induced Ulcerative Colitis via immunomodulation and gut microbiota reconfiguration

**DOI:** 10.1371/journal.pntd.0014340

**Published:** 2026-05-13

**Authors:** Lei Zhang, Lilin Li, Le Sun, Danhong Cheng, Shuwen Yang, Yu Wang, Xing Yan, Xiaoyan Zhu, Huan Zhang, Cuiying Li, Weixun Chunyu

**Affiliations:** 1 Department of Pathogen Biology and Immunology, School of Basic Medicine, Kunming Medical University, Kunming, Yunnan, PR China; 2 School of Forensic Medicine, Kunming Medical University, Kunming, Yunnan, PR China; 3 Department of Anesthesiology, The First Affiliated Hospital of Kunming Medical University, Kunming, Yunnan, PR China; Instituto de Salud Carlos III, SPAIN

## Abstract

Ulcerative colitis (UC) is a chronic inflammatory bowel illness with few treatment options, which means that new ways to treat it are needed. This study examined the protective effects and mechanisms of *Paragonimus proliferus* metacercaria-derived antigens (PmAg) in a dextran sulfate sodium (DSS)-induced mouse ulcerative colitis model. We discovered that intraperitoneal delivery of PmAg substantially mitigated colitis severity, as demonstrated by decreased weight loss, lower disease activity index scores, maintained colon length, and enhanced histopathological findings. Mechanistically, PmAg inhibited pro-inflammatory cytokines (IL-1β, TNF-α), increased the anti-inflammatory cytokine IL-10, and bolstered antioxidant defenses (SOD, GSH). It also restored the integrity of the intestinal barrier by boosting the number of goblet cells and the expression of tight junction proteins (Occludin, Claudin-1), while stopping the activation of the nuclear factor-kappa B (NF-κB) signaling pathway. Moreover, 16S rRNA sequencing demonstrated that PmAg reinstated gut microbiota α-diversity, diminished pathogenic genera (e.g., *Escherichia-Shigella*), and enhanced beneficial taxa (e.g., *Lachnospiraceae_NK4A136_group* and *Alistipes*). Integrated fecal metabolomics research revealed that PmAg altered metabolic profiles, specifically as significantly enriched the primary bile acid biosynthesis pathway, alpha-Linolenic acid metabolism pathway and Ubiquinone and other terpenoid-quinone biosynthesis pathways. In conclusion, our results suggested that PmAg could mitigates experimental colitis in mice by anti-inflammatory, improving gut microbiota and modulating fecal metabolomics.

## Author summary

Parasitic helminths have co-evolved with their hosts and developed sophisticated strategies to modulate host immune responses, which has attracted increasing interest for the treatment of inflammatory diseases. Ulcerative colitis is a chronic inflammatory bowel disease characterized by dysregulated immune responses, intestinal barrier damage, and alterations in gut microbiota, yet current treatments are often limited by side effects or loss of efficacy.In this study, we investigated whether antigens derived from the metacercariae of *Paragonimus proliferus* could alleviate experimental colitis in mice. Using a dextran sulfate sodium (DSS)–induced colitis model, we found that treatment with *P. proliferus* metacercaria-derived antigens significantly reduced disease severity, intestinal inflammation, and oxidative stress. These protective effects were associated with suppression of inflammatory signaling pathways, preservation of the intestinal epithelial barrier, partial restoration of gut microbial diversity, and changes in fecal metabolic profiles. Together, our findings reveal that *P. proliferus*–derived antigens exert multifaceted protective effects in experimental colitis. This study provides preliminary evidence supporting the potential of helminth-derived molecules as candidates for future investigation in immune-mediated intestinal diseases.

## Introduction

Ulcerative colitis (UC) is a long-term inflammatory bowel disease, characterized by recurrent attacks. It causes widespread inflammation of the colon’s mucosa. It is a serious medical problem with few treatment choices. Current primary therapies, such as aminosalicylates, corticosteroids, and biologics, either provide inadequate responses or are linked to significant adverse effects and diminishing efficacy over time [1,2]. In recent years, the “hygiene hypothesis” and related epidemiological studies have generated significant interest in helminth-derived immunomodulatory chemicals as prospective therapeutics for autoimmune and inflammatory illnesses [3,4]. The parasites have co-evolved with their hosts, creating advanced strategies to mitigate overwhelming immune responses and establish persistent, yet tolerable, infections [5–7]. The inherent immunoregulatory properties of helminth-derived antigens render them a viable yet underinvestigated source for the development of innovative anti-inflammatory biologics [8,9].

Studies on helminth therapy have shown that contact with specific parasites or their secreted/excreted substances can reduce the severity of diseases in animal models of asthma, multiple sclerosis, and inflammatory bowel disease [5,10,11]. The suggested processes frequently encompass the activation of regulatory T cells (Tregs), alternatively activated macrophages, and the synthesis of anti-inflammatory cytokines such as IL-10 and TGF-β, thus reinstating immunological homeostasis [12–14]. Nonetheless, the domain continues to be riddled with substantial knowledge deficiencies. Most research has concentrated on a restricted selection of model organisms such as *Heligmosomoides polygyrus*, *Schistosomasp*. or *Trichinella spiralis*, whereas the therapeutic potential of antigens from several other helminth species remains predominantly unexplored [13,15]. As a food-borne trematode, *Paragonimus sp.* has evolved unique adaptive strategies to establish persistent colonization in the host. During infection, the parasite must overcome the host’s mucosal immune surveillance to avoid clearance, and its metacercaria-derived antigens are speculated to play a pivotal role in this process-specifically by simulating “host immune tolerance signals”. Notably, this study is the first to discover that this evolutionary-adapted immunomodulatory strategy of *Paragonimus sp.* can be repurposed to alleviate the immune dysregulation in UC. More importantly, even though immune regulation is a major emphasis, the possible effects of these medicines on other important parts of UC pathogenesis, such the gut microbiota and host metabolism, have not been studied enough. This signifies a significant research deficiency, as dysbiosis of the gut microbiome and related metabolic abnormalities are now acknowledged as primary contributors to intestinal inflammation and barrier dysfunction [16,17].

This work examines the protective benefits of metacercaria-derived antigens from *Paragonimus proliferus* (PmAg), a food-borne trematode, against dextran sulfate sodium (DSS)-induced ulcerative colitis in mice, therefore addressing existing research gaps. We postulate that PmAg [18], in addition to its direct immunomodulatory actions, may provide protection by reestablishing gut microbial ecology and correcting host metabolic pathways, thereby functioning through a multifaceted mechanism. The reason for this work is predicated on the hypothesis that a comprehensive therapeutic strategy addressing the interrelated trio of immunological dysregulation, microbial imbalance, and metabolic dysfunction may achieve greater efficacy than approaches concentrating on a singular pathway.

To thoroughly evaluate this idea, we utilized a multi-omics and integrative analytical approach. The experimental approach used a validated DSS-induced mouse model of acute colitis to assess the macroscopic and histological efficacy of PmAg. We evaluate its effects on systemic and local inflammatory cytokine profiles, oxidative stress indicators, and the integrity of the intestinal epithelial barrier, including the expression of essential tight junction proteins at the molecular level [19]. We investigate the activation status of the nuclear factor-kappa B (NF-κB) signaling pathway, which is a key controller of pro-inflammatory gene expression [20]. Crucially, to move beyond purely host-centric mechanisms, we integrate 16S ribosomal RNA gene sequencing for gut microbiota profiling and non-targeted fecal metabolomics. This dual-omics approach allows for an unprecedented systems-level analysis, enabling us to delineate PmAg-induced shifts in microbial community structure and function and to identify associated changes in the host-metabolite landscape.

Therefore, the primary objective of this study is to determine whether administration of PmAg alleviates experimental UC and to elucidate the underlying mechanisms. We specifically seek to examine whether the protective effects are facilitated by the inhibition of pro-inflammatory signaling, the augmentation of antioxidant defenses, the maintenance of intestinal barrier integrity, and-novelty-through advantageous remodeling of the gut microbiota and related metabolic pathways. This study aims to identify causal relationships between PmAg-induced microbial alterations and host physiological effects by connecting microbial taxa with inflammatory, oxidative, and metabolic markers, thereby providing a more comprehensive knowledge of its therapeutic potential. The results of this study may facilitate the creation of innovative helminth-derived immunomodulatory drugs with multifunctional effects for the management of ulcerative colitis.

## Materials and methods

### Ethics statement

All animal experiments were conducted in strict accordance with the Guidelines for the Care and Use of Laboratory Animals and were approved by the Ethics Committee of Kunming Medical University (Approval No. KMMU20241526). All efforts were made to minimize animal suffering and to reduce the number of animals used.

### Preparation of *Paragonimus proliferus* Metacercaria-Derived Antigens (PmAg)

**Fig 1:**
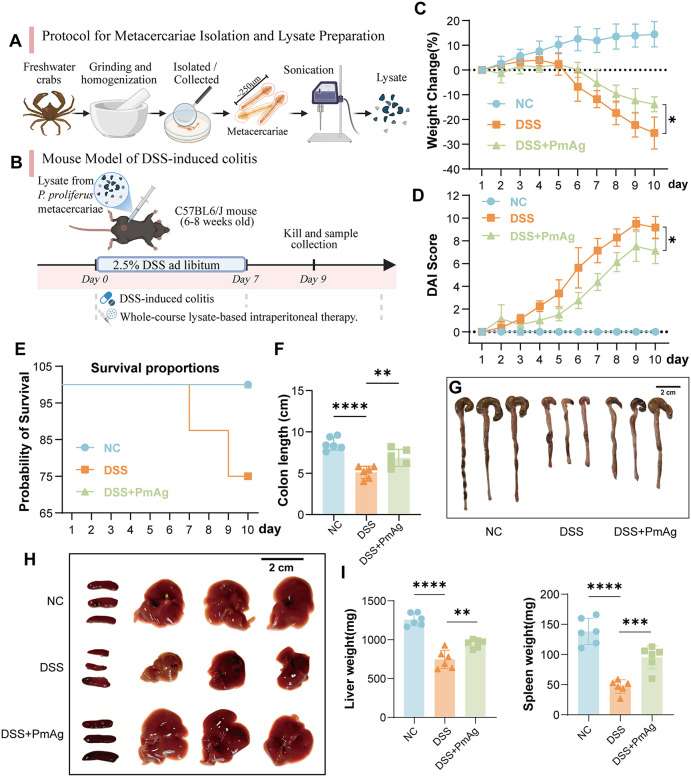
Experimental design and therapeutic effects of PmAg in DSS-induced colitis. **(A)** Schematic illustration of metacercaria isolation and preparation of *P. proliferus* metacercaria-derived antigen (PmAg). **(B)** Experimental protocol for DSS-induced colitis and PmAg intervention. C57BL/6J mice received 2.5% DSS to induce colitis and were treated with intraperitoneal PmAg; samples were collected at the end of the experiment. **(C)** Percentage change in body weight during the experimental period. **(D)** Disease activity index (DAI) scores assessed daily to evaluate colitis severity. **(E)** Survival curves of mice in each group. **(F)** Colon length measured at sacrifice. **(G)** Representative gross images of colons from each group. **(H)** Representative gross images of liver and spleen from each group. **(I)** Liver and spleen weights reflecting systemic inflammatory status. Data are presented as mean ± SD. *P < 0.05, **P < 0.01, ***P < 0.001, ****P < 0.0001. The elements of A and B were created in BioRender. Zhang, **L.** (2026) https://BioRender.com/1tmovmq.

Metacercariae were isolated from freshwater crabs, the second intermediate host of Paragonimus proliferus, collected from an endemic area in Xishuangbanna Autonomous Prefecture, Yunnan Province, China. A total of more than 300 metacercariae were obtained from 20 crabs, and these metacercariae were used for PmAg preparation. For antigen preparation, 300 metacercariae were washed several times with phosphate-buffered saline (PBS), thoroughly ground in liquid nitrogen, and homogenized in microcentrifuge tubes until no visible tissue fragments remained. The homogenate was then adjusted to a final volume of 10 mL with PBS and subjected to complete disruption using an ultrasonic cell disruptor, followed by centrifugation at 3000 × g for 1 h at 4 °C. The supernatant was collected as the PmAg preparation. The preparation process is shown in Fig 1A. The total protein concentration of PmAg was determined using a Pierce BCA Protein Assay Kit (Thermo Fisher Scientific, Rockford, IL, USA), yielding a final protein concentration of 1.27 μg/μL.Endotoxin levels were quantified, showing residual endotoxin of approximately 0.1 EU/mL. The PmAg was aliquoted and stored at −80 **°**C until use.

### Animals and DSS-induced ulcerative colitis model

All animal experiments were approved by the Ethics Committee of Kunming Medical University (Project No. KMMU20241526). Six-week-old male C57BL/6 mice (22 ± 2 g) were purchased from SPF (Beijing) Laboratory Animal Technology Co., Ltd. and housed in a specific pathogen-free facility.

After one week of acclimatization, 24 mice were randomly divided into three groups (n = 8/group): Normal control (NC), DSS model (DSS), DSS + PmAg (DSS + PmAg). Ulcerative colitis was induced by administering 2.5% (w/v) dextran sulfate sodium (DSS) in drinking water for 7 consecutive days, followed by 2 days of sterile water. Mice in the DSS + PmAg group received daily intraperitoneal injections of 100 μL PBS containing PmAg (approximately 127 µg of total protein) from day 0 to day 9. NC and DSS groups received equal volumes of PBS. All mice had free access to food and water throughout the experiment. The animal experiment procedure is shown in Fig 1B.

### Disease Activity Index (DAI) Assessment

Body weight, stool consistency, and fecal occult blood were recorded daily. The disease activity index (DAI) was calculated as the sum of scores for weight loss, stool consistency, and fecal bleeding, with a maximum score of 10. Detailed scoring criteria are provided in Table **A** in S1 Appendix.

### Sample collection

All mice were euthanized on day 10 after overnight fasting. Blood samples were collected for serum separation and ELISA analysis. Livers and spleens were dissected, and their weights were measured and recorded. Colon length from the cecum to the anus was measured. Colonic contents were collected for 16S rRNA sequencing and fecal metabolomics analysis. Colon tissues were harvested for histopathology, protein extraction, and RNA isolation.

### Histopathological and PAS staining

Colon tissues were fixed in 4% paraformaldehyde, paraffin-embedded, and sectioned. Hematoxylin and eosin (H&E) staining was conducted to assess tissue damage and inflammation, which were scored on a 0–4 scale according to four parameters (inflammatory cell infiltration, crypt architecture alteration, mucosal damage depth, and edema; criteria detailed in Table **B** in S1 Appendix). Goblet cell function was evaluated by periodic acid-Schiff (PAS) staining. The average number of goblet cells in 20 crypts per section was quantified.

### Enzyme-Linked Immunosorbent Assay (ELISA)

Serum levels of L-1β (interleukin-1β), TNF-α (tumor necrosis factor alpha), IL-10 (interleukin-10), SOD (superoxide dismutase) and GSH (glutathione peroxidase) were measured using commercial ELISA kits according to the manufacturer’s instructions (Jingmei Biological Technology Co., Ltd., Jiangsu, China). Absorbance was measured at 450 nm, and values were corrected by subtracting the negative control.

### Western blot analysis

Colon tissues were lysed in RIPA buffer supplemented with 1 mM PMSF (Solarbio). Equal amounts of protein were separated by SDS-PAGE and transferred to PVDF membranes (Millipore, Cat. No.: ISEQ00010). Membranes were blocked with 5% non-fat dry milk (dissolved in TBST containing 0.1% Tween-20) at room temperature for 1 h to reduce non-specific binding. After blocking, membranes were incubated overnight at 4 °C with the following primary antibodies: anti-Claudin 1 (Affinity, Cat. No.: DF6919, 1:2000 dilution), anti-Occludin (Proteintech, Cat. No.: 66378–1-Ig, 1:10000 dilution), anti-IκBα (Proteintech, Cat. No.: 10268–1-AP, 1:10000 dilution), anti-phosphorylated IκBα (p-IκBα; Invitrogen, Cat. No.: PA5–104851, 1:1000 dilution), anti-p65 (Invitrogen, Cat. No.: PA5–16545, 1:200 dilution), anti-phosphorylated p65 (p-p65; Invitrogen, Cat. No.: PA5–37718, 1:1000 dilution), and anti-β-actin (Invitrogen, Cat. No.: MA5–42946, 1:50000 dilution).

Subsequently, membranes were washed three times with TBST (5 min per wash) and incubated with HRP-conjugated secondary antibodies (Goat anti-Mouse IgG, Invitrogen, Cat. No.: 31431, 1:10000 dilution; Goat anti-Rabbit IgG, Invitrogen, Cat. No.: 31460, 1:10000 dilution) at room temperature for 1 h. After another three washes with TBST, protein signals were visualized using an enhanced chemiluminescence (ECL) detection reagent (Biosharp, Cat. No.: BL520B) and imaged with a Bio-Rad ChemiDoc XRS+ chemiluminescence imaging system. Band intensities were quantified using ImageJ software, and the relative expression levels of target proteins were normalized to that of β-actin.

### Immunohistochemistry (IHC)

Paraffin-embedded colon tissue sections (5 μm thick) were deparaffinized in xylene and rehydrated through a graded ethanol series (100%, 95%, 85%, 75%, 50%) to distilled water. Antigen retrieval was performed by boiling the sections in 0.01 M sodium citrate buffer (pH 6.0) for 20 min in a microwave oven, followed by natural cooling to room temperature. Endogenous peroxidase activity was quenched with 3% hydrogen peroxide solution at room temperature for 15 min. After three washes with phosphate-buffered saline (PBS, pH 7.4; 5 min per wash), sections were blocked with 5% normal goat serum at 37 °C for 30 min to eliminate non-specific antibody binding.

Subsequently, sections were incubated overnight at 4 °C with the following primary antibodies: anti-Occludin (Proteintech, Cat. No.: 66378–1-Ig, 1:250 dilution), anti-Claudin 1 (Affinity, Cat. No.: DF6919, 1:200 dilution), anti-IκBα (Proteintech, Cat. No.: 10268–1-AP, 1:3000 dilution), anti-phosphorylated p65 (p-p65; Invitrogen, Cat. No.: PA5–37718, 1:200 dilution), and anti-IL-1β (Proteintech, Cat. No.: 82191–3-RR, 1:200 dilution). After three PBS washes, sections were incubated with biotin-conjugated secondary antibodies (Maixin Bio SP Kit, Cat. No.: KIT-5020) at 37 °C for 30 min. Immunoreactive signals were visualized using 3,3’-diaminobenzidine (DAB) chromogen (included in the kit), with color development monitored under a light microscope and terminated by rinsing with distilled water after 3–5 min. Nuclei were counterstained with hematoxylin for 2 min, followed by differentiation in 1% hydrochloric acid-ethanol and rinsing with running tap water for 10 min. Finally, sections were dehydrated, cleared in xylene, and mounted with neutral balsam. The slice images were collected by the imaging system software. Image J 8.0 was used to calculate the relative expressions.

### RNA Extraction and Quantitative Real-Time PCR

Total RNA was extracted from mouse colon tissues using RNAiso Plus (Takara, Cat. No. 9108) according to the manufacturer’s instructions. RNA concentration and purity were assessed spectrophotometrically, and equal amounts of RNA were reverse-transcribed into cDNA using the RevertAid First Strand cDNA Synthesis Kit (Thermo Scientific, Cat. No. K1622). Quantitative real-time PCR was performed on a real-time PCR detection system using gene-specific primers. Primer sequences are provided in Table **C** in S1 Appendix. Gene expression levels were normalized to housekeeping genes and calculated using the 2^⁻ΔΔCt^ method.

### 16S rRNA sequencing analysis of gut microbiota

Total genomic DNA of the microbial community in fecal samples from each group was extracted using the FastPure Stool DNA Isolation Kit (MJYH, Shanghai, China) following the manufacturer’s instructions. The V3-V4 variable region of the 16S rRNA gene was amplified with specific primers: 338F (5′-ACTCCTACGGAGGCAGCA-3′) and 806R (5′-GGACTACHVGGGTWTCTAAT-3′). High-throughput sequencing of the amplified fragments was performed on the Illumina MiSeq PE 300 platform (Majorbio Bio-Pharm Technology Co., Ltd., Shanghai, China).

Paired-end raw reads were quality-filtered using fastp software [21](https://github.com/OpenGene/fastp, version 0.19.6) and spliced with FLASH software [22] (http://www.cbcb.umd.edu/software/flash, version 1.2.11). Denoising of the optimized sequences (after quality control and splicing) was conducted using the DADA2 plugin [23] in the QIIME2 workflow [24] to obtain amplicon sequence variants (ASVs). Taxonomic classification of ASVs was performed with the Naive Bayes classifier in QIIME2 based on the Silva 16S rRNA gene database (version 138).

Sobs index, ACE index, Shannon index and Simpson index were calculated to reveal the Alpha (α-) diversity of gut microbial community. Beta (β-) diversity was estimated using the Bray-Curtis distance, and analysis of similarities (ANOSIM) was used to determine differences in microbial communities among the three groups. β-diversity was visualized using principal coordinate analysis (PCoA) and non-metric multidimensional scaling (NMDS).

Core microorganisms in each group were identified by linear discriminant analysis effect size (LEfSe) with the criteria of LDA > 4 and *P* < 0.05.

### Fecal metabolomics analysis

Fecal samples (100 mg) were weighed into EP tubes and mixed with 800 μL of extraction solution (methanol:water = 4:1, v/v) containing 4 internal standards. Samples were ground thoroughly, and the supernatant was collected and transferred to analytical vials after centrifugation. Metabolite detection was performed using a UHPLC-Orbitrap Exploris 240 system (Thermo Fisher Scientific, Waltham, MA, USA) equipped with an HSS T3 chromatographic column (100 mm × 2.1 mm, 1.8 μm; Waters, Milford, MA, USA). Mass spectral signals were acquired in both positive and negative ion scanning modes with a mass range of m/z 70–1050.

Raw LC-MS data were imported into Progenesis QI software (Waters Corporation, Milford, MA, USA) for preprocessing (peak alignment, filtering, and normalization). Metabolite identification was achieved by matching mass spectral information with public metabolic databases. After preprocessing the data matrix, principal component analysis (PCA) and orthogonal partial least squares discriminant analysis (OPLS-DA) were performed using the ropls package (Version 1.6.2) in R language [25]. Significantly differential metabolites were screened based on variable importance in projection (VIP) values from the OPLS-DA model (VIP > 1) and Student’s t-test results (*P* < 0.05). Functional annotation of differential metabolites and enrichment analysis of metabolic pathways were conducted using the KEGG database (https://www.kegg.jp/kegg/pathway.html).

### Statistical analysis

All statistical analyses were performed using SPSS software (Version 21.0; SPSS Inc., Chicago, IL, USA). Multiple group comparisons were analyzed by one-way analysis of variance (ANOVA) followed by Tukey’s post-hoc test. Differences in gut microbiota between groups were determined using the Wilcoxon rank-sum test. Spearman’s correlation coefficients were calculated to generate correlation matrices between gut microbiota, biochemical parameters, and metabolites. All data are expressed as mean ± standard deviation (SD), and statistical significance was set at *P* < 0.05.

## Results

### PmAg Ameliorated DSS-induced ulcerative colitis in mice

Intraperitoneal administration of PmAg did not induce observable allergic reactions or adverse effects in mice throughout the experiment. Mice in the NC group exhibited a continuous increase in body weight. In contrast, DSS administration caused a significant and sustained body weight loss beginning on day 5. PmAg treatment markedly attenuated DSS-induced weight loss (Fig 1C). DAI scores in the DSS group increased significantly from day 3 onward, whereas mice receiving PmAg exhibited delayed and attenuated increases in DAI scores. By the end of the experiment, the DAI score in the DSS + PmAg group was significantly lower than that in the DSS group (Fig 1D), indicating that PmAg alleviated DSS-induced diarrhea and fecal bleeding. In addition, DSS exposure led to increased mortality and significant colon shortening. PmAg administration reduced mortality and significantly preserved colon length compared with the DSS group (Fig 1E-G). Compared with the DSS group, the liver and spleen weights in the DSS + PmAg group were significantly recovered (Fig 1H-I). The underlying mechanism might be associated with the ability of PmAg to alleviate intestinal mucosal injury, suppress systemic inflammatory responses triggered by DSS, and improve nutrient absorption and metabolic homeostasis in the host, thereby mitigating the weight loss of these vital organs. Collectively, these results demonstrate that PmAg exerts a protective effect against DSS-induced ulcerative colitis.

### PmAg suppressed Pro-inflammatory cytokine production and oxidative stress

**Fig 2:**
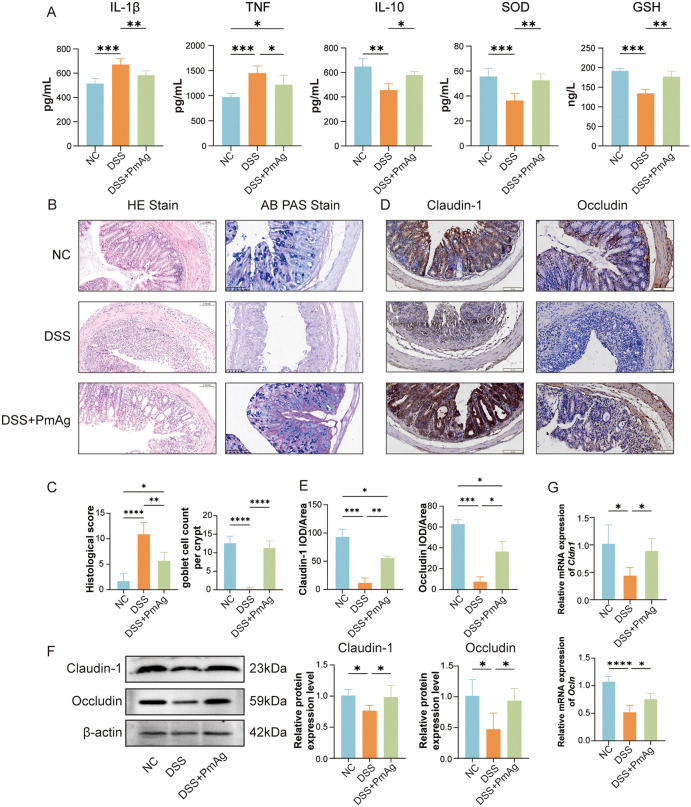
PmAg attenuates colonic inflammation, oxidative stress, and epithelial barrier dysfunction in DSS-induced colitis. **(A)** Levels of pro-inflammatory cytokines (IL-1β, TNF), anti-inflammatory cytokine (IL-10), and antioxidant indicators (SOD and GSH) in colonic tissues from NC, DSS, and DSS + PmAg groups. **(B)** Representative hematoxylin-eosin (H&E) staining showing colonic histopathological alterations and Alcian blue-periodic acid-Schiff (AB-PAS) staining showing goblet cell abundance in each group. **(C)** Quantification of histological scores and goblet cell numbers based on staining in panel **B. (D)** Immunohistochemical staining of the tight junction proteins Claudin-1 and Occludin in colonic sections. **(E)** Quantification of Claudin-1- and Occludin-positive staining areas corresponding to panel **D. (F)** Western blot analysis of Claudin-1 and Occludin expression in colonic tissues. **(G)** Relative mRNA expression levels of tight junction-related genes in colonic tissues. The mRNA expression levels were determined by qRT-PCR and normalized to GAPDH using the 2^-ΔΔCt method.Data are presented as mean ± SD. *P < 0.05, **P < 0.01, ***P < 0.001, ****P < 0.0001.

To investigate the anti-inflammatory effects of PmAg, serum levels of cytokines were measured. Compared with the NC group, DSS-treated mice exhibited significantly elevated levels of the pro-inflammatory cytokines IL-1β and TNF-α, accompanied by a marked reduction in the anti-inflammatory cytokine IL-10. PmAg treatment significantly reversed these DSS-induced cytokine alterations (Fig 2A).

Given the close association between oxidative stress and UC progression, antioxidant parameters were further assessed. DSS administration resulted in a significant decrease in SOD and GSH levels. Notably, PmAg treatment markedly restored both SOD and GSH levels compared with the DSS group (Fig 2A). These findings indicate that PmAg alleviates DSS-induced colitis by suppressing inflammatory responses and enhancing antioxidant defense mechanisms.

### PmAg Attenuated DSS-induced histopathological damage and preserved intestinal barrier integrity

Histological examination by H&E staining revealed that colonic tissues from NC mice exhibited intact epithelial architecture, well-organized crypt structures, abundant goblet cells, and minimal inflammatory infiltration. In contrast, DSS-treated mice displayed severe epithelial disruption, crypt destruction, and extensive inflammatory cell infiltration. PmAg administration markedly alleviated these pathological changes, showing improved epithelial integrity and reduced inflammatory infiltration (Fig 2B, C).PAS staining demonstrated a significant reduction in goblet cell numbers in DSS-treated mice, whereas PmAg treatment significantly increased goblet cell density, suggesting enhanced mucosal protection and epithelial repair capacity (Fig 2B, C).

To further assess intestinal barrier integrity, the expression of tight junction proteins was examined. Immunohistochemical analysis showed reduced staining intensity of these tight junction proteins in the DSS group, which was preserved following PmAg treatment (Fig 2D, E). Consistently, Western blot analysis revealed that DSS significantly reduced the protein expression of Occludin and Claudin-1, while PmAg treatment markedly restored their expression levels (Fig 2F). qRT-PCR analysis further confirmed that PmAg significantly upregulated the mRNA expression of these barrier-associated genes, *including Ocln and Cldn1.* (Fig 2G). Together, these results demonstrate that PmAg protects intestinal barrier integrity in DSS-induced colitis.

### PmAg Suppressed DSS-Induced NF-κB signaling pathway activation

**Fig 3:**
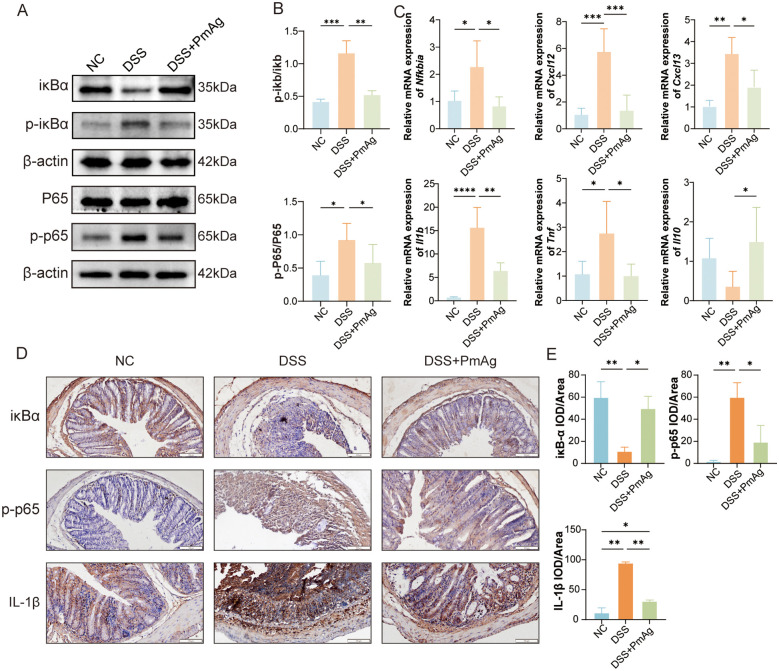
PmAg suppresses NF-κB signaling and downstream inflammatory responses in DSS-induced colitis. **(A)** Representative Western blot images showing the protein expression of IκB, phosphorylated IκB (p-IκB), NF-κB p65, phosphorylated p65 (p-p65), and β-actin in colonic tissues from Normal Control (NC), DSS, and DSS + PmAg groups. **(B)** Quantification of the ratios of p-IκB/IκB and p-p65/p65 based on densitometric analysis of Western blot results in panel **A. (C)** Relative mRNA expression levels of NF-κB-related genes and inflammatory cytokines (*Nfkbia*, *Cxcl12*, *Cxcl13*, *Il1b*,*Tnf, and Il10*) in colonic tissues. The mRNA expression levels were determined by qRT-PCR and normalized to GAPDH using the 2^-ΔΔCt method. **(D)** Representative immunohistochemical staining of IκB, p-p65, and IL-1β in colon sections from each group. **(E)** Quantification of immunohistochemical staining intensity (IOD/area) corresponding to panel **D.** Data are presented as mean ± SD. *P < 0.05, **P < 0.01, ***P < 0.001.

To elucidate the molecular mechanisms underlying PmAg’s anti-inflammatory effects, key components of the NF-κB signaling pathway were assessed in NC, DSS, and DSS + PmAg groups. Western blot analysis (Fig 3A) showed that DSS treatment notably elevated the phosphorylation levels of IκBα (p-IκBα) and p65 (p-p65)-hallmarks of NF-κB activation-while PmAg administration markedly reduced these phosphorylated proteins. Quantitative densitometric analysis (Fig 3B) further confirmed that DSS significantly increased the p-IκBα/IκBα and p-p65/p65 ratios, which were reversed by PmAg. We further assessed the mRNA expression levels of inflammation-related genes qRT-PCR (Fig 3C, D). DSS treatment significantly upregulated the mRNA expression of pro-inflammatory mediators (*Nfkbia*, *Cxcl12*, *Cxcl13*, *Il1b*, *Tnf*), while PmAg suppressed this DSS-induced upregulation. In contrast, the reduced *Il10* mRNA level in DSS-treated mice was partially restored following PmAg administration. IHC staining (Fig 3E) and quantitative analysis (Fig 3F) validated these findings: DSS-treated mice exhibited enhanced nuclear localization of p-p65, intense IL-1β staining, and diminished IκBα signals in colon tissues. PmAg treatment substantially attenuated p-p65 nuclear accumulation and IL-1β expression, while restoring IκBα staining. Collectively, these results suggest that the protective effects of PmAg against DSS-induced colonic inflammation are associated, at least partially, by suppression of NF-κB signaling pathway activation.

### PmAg beneficially modulated gut microbiota composition in DSS-induced colitis

**Fig 4:**
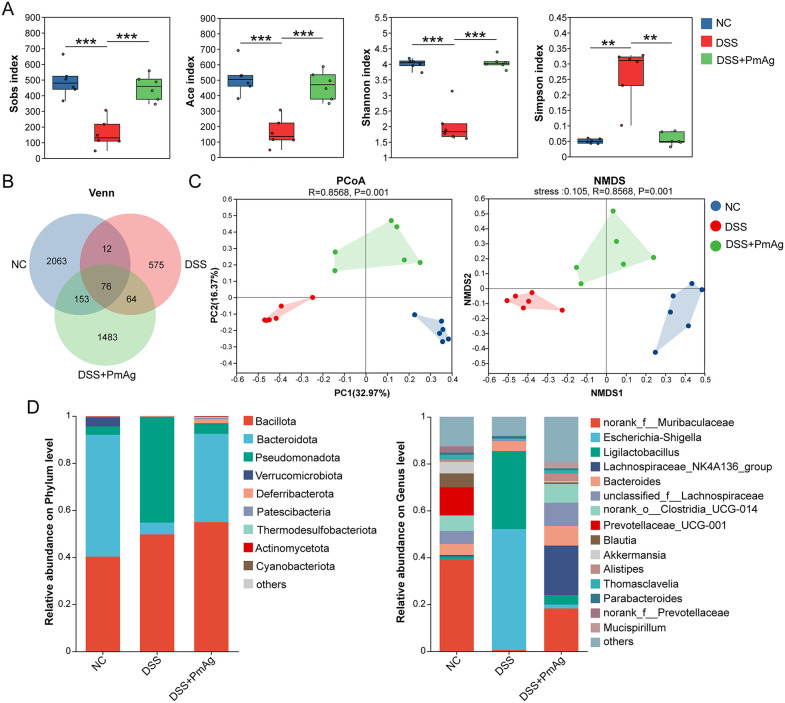
PmAg reshapes gut microbiota composition and diversity in DSS-induced colitis. **(A)** Alpha diversity indices of the gut microbiota, including Sobs, ACE, Shannon, and Simpson indices, in Normal Control (NC), DSS-induced colitis (DSS), and DSS + PmAg groups. **(B)** Venn diagram showing shared and unique operational taxonomic units (OTUs) among the three groups. **(C)** Beta diversity analysis based on principal coordinate analysis (PCoA; Bray-Curtis distance) and non-metric multidimensional scaling (NMDS), demonstrating distinct microbial community structures among groups. **(D)** Relative abundance of gut microbiota at the phylum (left) and genus (right) levels in each group. Data are presented as mean ± SD. *P < 0.05, **P < 0.01, ***P < 0.001.

To explore whether PmAg modulates gut microbiota composition, 16S rRNA gene sequencing was performed on colonic contents. Rarefaction curve analysis indicated that sequencing depth was sufficient to capture the majority of microbial diversity in each sample (Fig A(A) in S1 Appendix). α-diversity analysis revealed that DSS significantly reduced microbial richness and diversity, as indicated by decreased Sobs index, ACE index, Shannon index, and increased Simpson index. PmAg treatment significantly restored these indices compared with the DSS group (Fig 4A). The Venn diagram indicated that the NC and DSS groups shared 88 ASVs, the NC and DSS + PmAg groups shared 229ASVs, the DSS and DSS + PmAg groups shared 140 ASVs, and all three groups shared 76 ASVs. On the other hand, the NC, DSS, and DSS + PmAg groups contained 2063, 575 and 1483 unique ASVs respectively (Fig 4B).

β-diversity analysis based on Bray-Curtis distance revealed clear separation among the NC, DSS, and DSS + PmAg groups (ANOSIM R = 0.8568, *P* = 0.001), indicating significant differences in overall microbial community structure (Fig 4C). DSS treatment induced a pronounced shift in gut microbiota composition, whereas PmAg administration reshaped the microbial community into a distinct and separate cluster.

Taxonomic profiling was subsequently performed at the phylum and genus levels. At the phylum level, nine phyla were identified across all samples, including Bacillota, Bacteroidota, Pseudomonadota, Verrucomicrobiota, Deferribacterota, Patescibacteria, Thermodesulfobacteriota, Actinomycetota, and Cyanobacteriota. Among these, Pseudomonadota exhibited the most prominent alteration, showing a marked increase in the DSS group compared with the NC group (44.91%), which was significantly reduced following PmAg intervention (9.11%) (Fig A(B) in S1 Appendix.

**Fig 5:**
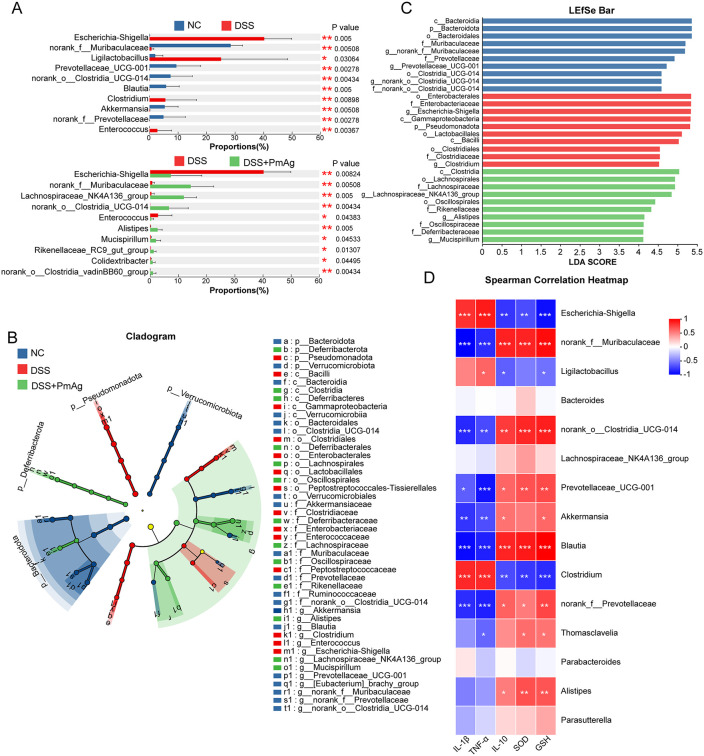
PmAg modulates disease-associated gut microbiota and their correlations with inflammatory and oxidative stress markers. **(A)** Differentially abundant bacterial genera between NC and DSS groups (upper panel) and between DSS and DSS + PmAg groups (lower panel), identified using the Wilcoxon rank-sum test. **(B)** LEfSe cladogram showing taxonomic differences among NC, DSS, and DSS + PmAg groups, with different colors indicating taxa enriched in each group. **(C)** LEfSe linear discriminant analysis (LDA) scores (LDA > 4.0) identifying key discriminatory taxa among groups. **(D)** Spearman correlation heatmap showing associations between the relative abundance of dominant bacterial genera and inflammatory cytokines (IL-1β, TNF-α, IL-10) and oxidative stress indicators (SOD and GSH). *P < 0.05, **P < 0.01, ***P < 0.001.

At the genus level, the relative abundances of the top 15 genera were compared among groups (Fig 4D). DSS exposure significantly increased the abundance of *Escherichia-Shigella*, *Ligilactobacillus*, *Clostridium*, and *Enterococcus*, while significantly decreasing norank_f__Muribaculaceae, *Prevotellaceae_UCG-001*, norank_o__Clostridia_UCG-014, *Blautia*, *Akkermansia*, and norank_f__Prevotellaceae relative to the NC group. PmAg treatment significantly reversed these DSS-induced alterations, characterized by reduced abundances of *Escherichia-Shigella* and *Enterococcus* and enrichment of norank_f__Muribaculaceae, *Lachnospiraceae_NK4A136_group*, norank_o__Clostridia_UCG-014, *Alistipes*, *Mucispirillum*, *Rikenellaceae_RC9_gut_group*, *Colidextribacter*, and norank_o__Clostridia_vadinBB60_group (Fig 5A).

To further identify UC-associated discriminatory taxa, LEfSe analysis was conducted using an LDA score cutoff of 4.0 (Fig 5B, C). The NC group was characterized by enrichment of *Bacteroidota*, norank_f__Muribaculaceae, *Prevotellaceae_UCG-001*, and norank_o__Clostridia_UCG-014. In contrast, the DSS group was dominated by pathogenic or potentially pathogenic taxa, including *Pseudomonadota*, *Escherichia-Shigella*, and *Clostridium*. Notably, the DSS + PmAg group was enriched in taxa generally considered beneficial, such as *Lachnospiraceae_NK4A136_group*, *Alistipes*, and *Mucispirillum*.

Spearman correlation analysis was performed to evaluate associations between the top 15 genera and inflammatory and oxidative stress-related parameters (Fig 5D). *Escherichia-Shigella* and *Clostridium* were positively correlated with pro-inflammatory cytokines IL-1β and TNF and negatively correlated with IL-10, SOD, and GSH. In contrast, norank_f__Muribaculaceae, norank_o__Clostridia_UCG-014, *Prevotellaceae_UCG-001*, *Blautia*, and norank_f__Prevotellaceae showed negative correlations with IL-1β and TNF and positive correlations with IL-10, SOD, and GSH. Notably, *Alistipes*, a key taxon enriched following PmAg intervention, was positively correlated exclusively with anti-inflammatory (IL-10) and antioxidant (SOD and GSH) markers.

### Correlation between gut microbiota and inflammatory and oxidative stress parameters

Spearman correlation analysis was performed to evaluate the relationships between the relative abundance of the 15 most abundant genera and biochemical parameters. Pro-inflammatory genera, including *Escherichia-Shigella* and *Clostridium*, were positively correlated with IL-1β and TNF-α and negatively correlated with IL-10, SOD, and GSH. In contrast, beneficial genera such as *norank_f__Muribaculaceae*, *norank_o__Clostridia_UCG-014*, *Prevotellaceae_UCG-001*, *Blautia*, and *norank_f__Prevotellaceae* exhibited opposite correlation patterns (Fig 5A).

Notably, *Alistipes*, a core genus enriched following PmAg treatment, showed significant positive correlations exclusively with IL-10, SOD, and GSH, highlighting its potential role in mediating the anti-inflammatory and antioxidant effects of PmAg.

### PmAg reshaped fecal metabolic profiles in DSS-induced colitis

**Fig 6:**
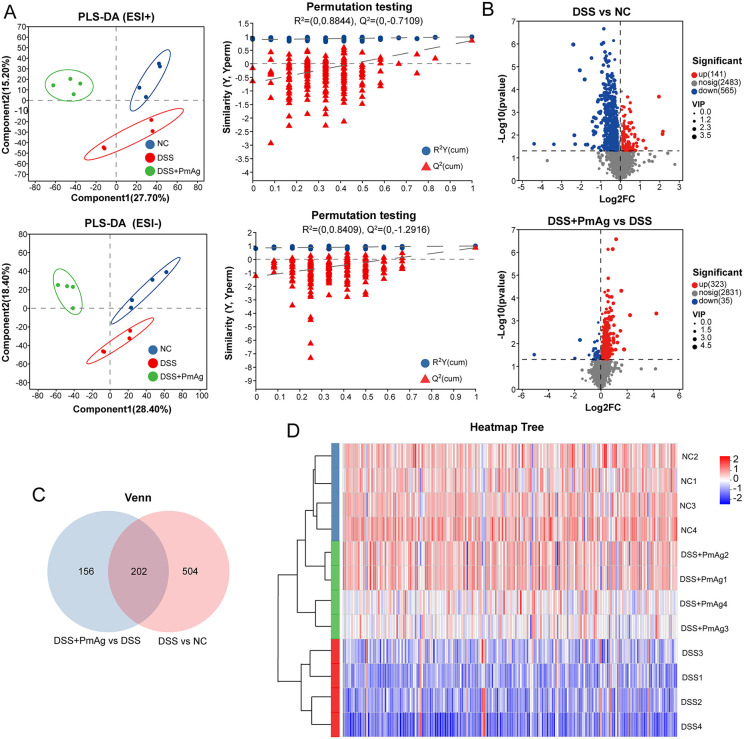
PmAg reshapes fecal metabolomic profiles in DSS-induced colitis. **(A)** Partial least squares-discriminant analysis (PLS-DA) score plots of fecal metabolomic profiles in positive (ESI⁺) and negative (ESI⁻) ion modes, showing clear separation among NC, DSS, and DSS + PmAg groups. Corresponding permutation tests validate the robustness of the PLS-DA models. **(B)** Volcano plots illustrating differential metabolites between DSS and NC groups (upper panel) and between DSS + PmAg and DSS groups (lower panel). Metabolites are colored according to significance and variable importance in projection (VIP) values. **(C)** Venn diagram showing the overlap of differential metabolites identified between DSS vs NC and DSS + PmAg vs DSS comparisons. **(D)** Hierarchical clustering heatmap of significantly altered metabolites across all samples, illustrating distinct metabolic patterns among groups.

**Fig 7:**
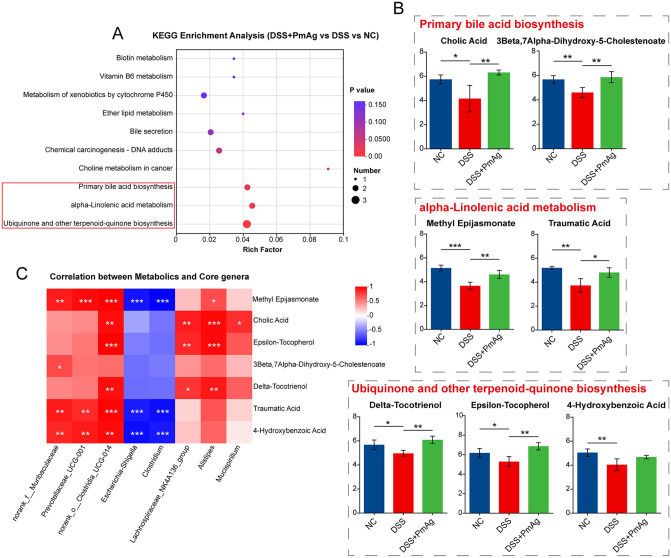
PmAg regulates bile acid, fatty acid, and antioxidant-related metabolic pathways in DSS-induced colitis. **(A)** KEGG pathway enrichment analysis of differential metabolites identified among DSS, DSS + PmAg, and NC groups. Pathways related to primary bile acid biosynthesis, α-linolenic acid metabolism, and ubiquinone and other terpenoid-quinone biosynthesis are highlighted. **(B)** Relative levels of representative metabolites involved in primary bile acid biosynthesis (cholic acid and 3β,7α-dihydroxy-5-cholestenoate), α-linolenic acid metabolism (methyl epijasmonate and traumatic acid), and ubiquinone and other terpenoid-quinone biosynthesis (δ-tocotrienol, ε-tocopherol, and 4-hydroxybenzoic acid) across groups. **(C)** Spearman correlation heatmap showing associations between key differential metabolites and core bacterial genera identified by LEfSe analysis. Data are presented as mean ± SD. *P < 0.05, **P < 0.01, ***P < 0.001.

To explore the effect of Pm on the fecal metabolic profile of UC mice, we analyzed the fecal metabolome of mice by non-targeted metabolomics method. The effects of PmAg on fecal metabolites of UC mice were investigated by PCA and Partial Least Squares Discriminant Analysis (PLS-DA). In both positive and negative ion modes, PCA analysis showed that the internal variation of each group was small, and the three groups of samples were clustered separately, and the difference in metabolic profiles between groups was significant (Fig B(A) in S1 Appendix). PLS-DA analysis also showed that there was a significant separation between the three groups of samples (cation model: R^2^ = 0.8844, Q^2^ = -0.7109; anion model: R^2^ = 0.08409, Q^2^ = -1.2916) (Fig 6A). In addition, we used PLS-DA analysis to compare the DSS group and the NC group, and the Pm group and the DSS group under the positive and negative ion models, respectively. The results showed that there were large differences between the groups (Fig B(B) in S1 Appendix). Based on the criteria |fold change (FC)| > 1, *P* < 0.05, and variable importance in projection (VIP) > 1.0, differential metabolites were identified. Volcano plots show the statistically significant differential metabolites between groups (Fig 6B). The results showed that there were 706 significantly altered metabolites between the DSS group and the NC group, including 141 up-regulated and 565 down-regulated. Compared with the DSS group, the DSS + PmAG group showed 358 significantly changed metabolites, including 323 up-regulated and 35 down-regulated. Venn analysis further revealed that 202 differential metabolites coexisting among the three groups were significantly changed in pairwise comparisons (Fig 6C). The relative abundance of these differential metabolites (DEMs) was analyzed by clustering. The results showed that the fecal metabolic profiles of the NC group and the DSS group were significantly different. The metabolic profiles of the PM group and the NC group were clustered into one cluster, indicating that the treatment partially restored the altered metabolic profiles to normal levels (Fig 6D). Pathway enrichment analysis based on the KEGG database was performed on these DEMs to determine their role in metabolic pathways. A total of 26 differential metabolites were identified, involving 24 metabolic pathways (Table 1). Fig 7A lists the top 10 significantly affected pathways. Pathways with a significance level of *P* < 0.05 were considered as potential targets for PmAg intervention in UC. These metabolites were related to Primary bile acid biosynthesis, alpha-Linolenic acid metabolism, and Ubiquinone and other terpenoid-quinone biosynthesis. To visualize these changes, we compared the intergroup differences in the content of seven metabolites involved in the above three synthetic pathways (Fig 7B). Compared with the DSS group, PmAg significantly upregulated Cholic Acid, 3Beta,7Alpha-Dihydroxy-5-Cholestenoate, Methyl Epijasmonate, Traumatic Acid, Delta-Tocotrienol and Epsilon-Tocopherol.

**Table 1:** KEGG pathway enrichment analysis of differential metabolites.

No.	Pathway Description	Ratio in study	Ratio in pop	*P* value	*P* adjust
1	Ubiquinone and other terpenoid-quinone biosynthesis	3/26	71/4660	0.006855	0.654
2	alpha-Linolenic acid metabolism	2/26	44/4660	0.02453	0.8824
3	Primary bile acid biosynthesis	2/26	47/4660	0.02775	0.8824
4	Choline metabolism in cancer	1/26	11/4660	0.05975	1
5	Chemical carcinogenesis - DNA adducts	2/26	78/4660	0.06941	1
6	Bile secretion	2/26	97/4660	0.101	1
7	Ether lipid metabolism	1/26	25/4660	0.1308	1
8	Metabolism of xenobiotics by cytochrome P450	2/26	121/4660	0.1454	1
9	Vitamin B6 metabolism	1/26	29/4660	0.1502	1
10	Biotin metabolism	1/26	29/4660	0.1502	1
11	Chemical carcinogenesis - receptor activation	1/26	29/4660	0.1502	1
12	Phenylalanine, tyrosine and tryptophan biosynthesis	1/26	35/4660	0.1784	1
13	Porphyrin metabolism	2/26	148/4660	0.1992	1
14	Fructose and mannose metabolism	1/26	55/4660	0.2662	1
15	Nicotinate and nicotinamide metabolism	1/26	55/4660	0.2662	1
16	Glycerophospholipid metabolism	1/26	56/4660	0.2704	1
17	Ascorbate and aldarate metabolism	1/26	57/4660	0.2745	1
18	Biosynthesis of cofactors	3/26	328/4660	0.2754	1
19	Folate biosynthesis	1/26	58/4660	0.2786	1
20	Nucleotide metabolism	1/26	58/4660	0.2786	1
21	Pyrimidine metabolism	1/26	66/4660	0.3106	1
22	Cysteine and methionine metabolism	1/26	68/4660	0.3183	1
23	Biosynthesis of unsaturated fatty acids	1/26	74/4660	0.3412	1
24	Arachidonic acid metabolism	1/26	79/4660	0.3597	1
25	ABC transporters	1/26	138/4660	0.5433	1

### Integrated correlation analysis of gut microbiota and differential metabolites

To further investigate the associations between gut microbiota and fecal metabolites, Spearman correlation analysis was performed between seven differential metabolites and eight bacterial genera identified by LEfSe analysis with an LDA score > 4. *Escherichia-Shigella* and *Clostridium* exhibited negative correlations with all seven metabolites and showed significant negative associations with methyl epijasmonate, traumatic acid, and 4-hydroxybenzoic acid. In contrast, norank_f__Muribaculaceae, *Prevotellaceae_UCG-001*, norank_o__Clostridia_UCG-014, *Lachnospiraceae_NK4A136_group*, *Alistipes*, and *Mucispirillum* were positively correlated with all seven metabolites (Fig 7C).

Collectively, these association patterns indicate that PmAg administration is accompanied by coordinated shifts in gut microbial taxa and fecal metabolic profiles, highlighting a close linkage between microbiota composition and metabolic alterations during the amelioration of DSS-induced colitis.

## Discussion

Ulcerative colitis (UC) is a long-term inflammatory bowel illness that causes inflammation of the mucosa that comes and goes, mostly in the colon and rectum [2]. Its pathophysiology include dysregulated immunological responses, oxidative stress, compromised intestinal barrier function, and gut microbiota dysbiosis [1,26,27]. Current therapeutic techniques, encompassing aminosalicylates, corticosteroids, and biologics, frequently produce poor results accompanied by considerable side effects, underscoring the pressing want for innovative therapeutic modalities [28]. Recent data indicates that immunomodulatory chemicals derived from helminths may hold potential for the treatment of autoimmune disorders by reestablishing immunological homeostasis [29,30]. Nonetheless, the exact mechanisms through which these compounds confer their protective benefits, especially via microbiome and metabolic regulation, are inadequately elucidated.

This study investigates the therapeutic potential of *Paragonimus proliferus* metacercaria-derived antigens (PmAg) in a dextran sulfate sodium (DSS)-induced murine UC model. Our findings show that PmAg treatment was associated with alleviates colitis severity by decreased inflammatory responses, improved antioxidant status, restoring gut barrier integrity, and suppression NF-κB signaling [31,32]. In addition, multi-omics analyses reveal coordinated alterations in reshapes gut microbiota composition and fecal metabolic profiles following PmAg administration. The following discussion focusing on the potential immunomodulatory effects of PmAg, its microbiome-metabolome crosstalk, and broader implications of parasite-derived molecules for UC therapy.

PmAg significantly suppressed the activation of the nuclear factor-kappa B (NF-κB) pathway, as evidenced by reduced phosphorylation of IκBα and p65, and diminished nuclear translocation of p-p65 [33]. These changes were accompanied by downregulation of pro-inflammatory mediators (IL-1β, TNF-α) and partial restoration of anti-inflammatory cytokine IL-10 [34,35]. Given the established role of NF-κB pathway as a central regulator of intestinal inflammation in UC, these findings support the possibility that suppression of NF-κB-related inflammatory signaling contributes, at least in part, to the protective effects of PmAg. In parallel, PmAg treatment restored superoxide dismutase (SOD) and glutathione (GSH) levels, suggesting an improvement in oxidative stress status. Although this may be consistent with potential involvement of antioxidant regulatory pathways such as nuclear factor erythroid 2-related factor 2 (Nrf2) pathway, and this possibility requires further investigation [1,36,37]. Collectively, these findings support an anti-inflammatory and anti-oxidative profile of PmAg in DSS-induced colitis. At the epithelial level, PmAg demonstrated remarkable efficacy in preserving intestinal barrier integrity, a critical factor in UC pathogenesis [38,39]. The restoration of tight junction proteins (Occludin, Claudin-1) and goblet cell density, as confirmed by immunohistochemistry and quantitative polymerase chain reaction (qPCR), suggests that PmAg promotes epithelial repair and mucosal healing. These effects may be mediated through the interplay between NF-κB suppression and altered microbiota-derived metabolites. In addition, the upregulation of barrier-associated genes indicates that PmAg may support epithelial repair processes and mucosal homeostasis. These observations align with emerging evidence that helminth-derived molecules can modulate epithelial barrier function and intestinal homeostasis, positioning PmAg as a promising candidate for barrier-targeted therapies in UC [40–42].Beyond its effects on host inflammatory responses, PmAg treatment was also associated with alterations in gut microbiota composition and host-microbiota metabolic interactions [43]. PmAg administration restored microbial α-diversity and shifted the community structure toward beneficial taxa commonly associated with intestinal health (e.g., *Lachnospiraceae_NK4A136_group*, *Alistipes*), while reducing the relative abundance of potentially pathogenic genera (e.g., *Escherichia-Shigella*) [16,43]. Spearman correlation analysis revealed strong associations between these microbial shifts and improved inflammatory and oxidative stress markers, suggesting a causal role for microbiota remodeling in PmAg’s therapeutic effects [44]. Previous studies on helminth-microbiota interactions have largely focused on changes induced by live parasite infection. Accumulating evidence suggests that helminths can reshape the intestinal microbial ecosystem during long-term host adaptation, often by increasing microbial diversity and selectively enriching taxa associated with immune homeostasis [45,46]. In this context, the PmAg-induced enrichment of beneficial taxa observed here appears directionally consistent with the broader concept that helminth-derived signals can promote microbiota configurations linked to reduced inflammation. For example, several studies on *Heligmosomoides polygyrus* (Hp) have reported an expansion of *Lactobacillus* [47,48], and Hp has also been shown to influence gut microbiota metabolism, specifically promoting the production of SCFAs, which may contribute to asthma treatment [49]. Similarly, late-stage *Trichinella* infection has been associated with increased abundance of probiotic *Lactobacillus*, potentially contributing to parasite-associated immune modulation [50]. The ES-62 derived from Acanthocheilonema viteae has been confirmed to be able to prevent arthritis and is related to the intestinal microbiota [51]. Together, these findings suggest that parasite-derived antigens may retain partial capacity to influence host microbial ecology and metabolic activity, thereby contributing, at least in part, to protection against inflammatory disease.

Notably, fecal metabolomics identified PmAg-induced restoration of bile acid and terpenoid-quinone biosynthesis pathways, which are critical for immune regulation and redox balance [52,53]. The integrated analysis of microbiota and metabolomic data underscores the potential of PmAg to synchronize host metabolic and microbial networks, offering a holistic approach to UC management [54]. These findings advance our understanding of helminth-mediated immunomodulation and its translational potential in inflammatory bowel diseases.

### Limitations and future directions

This study offers significant evidence regarding the therapeutic efficacy of *Paragonimus proliferus* metacercaria-derived antigens (PmAg) in DSS-induced colitis; however, several limitations of this study should be acknowledged. First, although PmAg treatment was associated with coordinated improvements in intestinal pathology, inflammatory responses, barrier-related markers, gut microbiota, and fecal metabolites, these findings remain primarily associative and do not establish direct causal mechanisms. Future studies using NF-κB inhibitors, pathway-targeted interventions, or genetically modified animal models will be valuable for strengthening causal inference regarding the therapeutic effects of PmAg. Second, while the endotoxin content of the PmAg preparation was low (approximately 0.1 EU/mL), and previous studies have suggested that low-level endotoxin contamination is unlikely to exert major immunological effects under similar conditions [55,56], a minor contribution from trace endotoxin cannot be completely excluded. However, given the low endotoxin level and the coordinated improvements observed in inflammatory, microbiota, and metabolomic outcomes, it is unlikely that endotoxin alone accounts for the overall protective effects of PmAg. Future studies incorporating endotoxin removal or neutralization strategies will help further clarify this issue [57]. Third, PmAg was evaluated as a crude metacercaria-derived antigen preparation, and the specific bioactive components responsible for its protective effects remain to be identified. Our future work should focus on enhancing the identification and evaluation of the activity of single components. In addition, a dedicated PmAg-only control group was not included in the current study. Future studies should focus on isolating defined parasite-derived molecules, clarifying their cell-specific targets and microbiota-related effects, and further evaluating their safety and therapeutic potential in translational settings, which may provide broader insight into the therapeutic potential of parasite-derived products for inflammatory diseases.

### Immune regulation of parasites

Parasitic helminths have co-evolved with their hosts over millions of years and developed highly efficient strategies to modulate host immune responses, thereby promoting parasite persistence while limiting excessive immunopathology [58]. In this context, the present study demonstrates that antigens derived from *Paragonimus proliferus* metacercariae exert robust protective effects in a DSS-induced colitis model, serving as a tractable system to interrogate helminth-mediated immunoregulation. Rather than acting through a single molecular target, PmAg elicited coordinated effects on inflammatory signaling, epithelial barrier integrity, gut microbiota composition, and associated metabolic pathways, reflecting the multifaceted nature of host-parasite interactions. Although the use of a complex antigenic mixture and the absence of causal validation between microbiota alterations and disease amelioration represent limitations of this study, our integrated multi-omics approach provides system-level insights into how helminth-derived antigens reshape the intestinal immune-microbiota axis. Importantly, this work does not advocate the therapeutic use of live parasites, but instead highlights the potential of parasite-derived molecules as a source of novel immunomodulatory strategies. Collectively, our findings advance the understanding of helminth-host immune interactions and underscore their relevance to inflammatory diseases with global health significance.

## Conclusion

In conclusion, this study provides proof-of-concept evidence that non-infectious, non-living antigens derived from *Paragonimus proliferus* metacercariae can alleviate DSS-induced colitis. PmAg treatment was associated with reduced inflammatory responses, improved intestinal barrier-related markers, and coordinated alterations in gut microbiota composition and fecal metabolic profiles. Integrated microbiome-metabolome analyses highlight the capacity of helminth-derived molecules to coordinately influence host immune responses and microbial ecology. Althoughthese findings do not support immediate clinical application, they establish a conceptual framework for the identification of bioactive parasite-derived immunomodulatory molecules and their future validation in human-relevant models.

## Supporting information

S1 Appendix Table A Disease activity index (DAI) scoring criteria. Table B. Histopathological scoring criteria. Table C. Primer sequences used for quantitative real-time PCR. Fig A. Intestinal microbiota diversity and phylum-level compositional changes in DSS-induced colitis following PmAg intervention. Fig B. Multivariate statistical analysis of metabolomic profiles in DSS-induced colitis following PmAg intervention.
(DOCX)
