# Supplementary material for: Paragonimus proliferus Metacercaria-derived antigens mitigate DSS-Induced Ulcerative Colitis via immunomodulation and gut microbiota reconfiguration
Source: PLoS Negl Trop Dis. 2026 May 13;20(5):e0014340. doi: 10.1371/journal.pntd.0014340 (PMC13197073; doi:10.1371/journal.pntd.0014340)
Supplement: S1 Appendix — Table A Disease activity index (DAI) scoring criteria. Table B. Histopathological scoring criteria. Table C. Primer sequences used for quantitative real-time PCR. Fig A. Intestinal microbiota diversity and phylum-level compositional changes in DSS-induced colitis following PmAg intervention. Fig B. Multivariate statistical analysis of metabolomic profiles in DSS-induced colitis following PmAg intervention. (DOCX) [file pntd.0014340.s001.docx]

Table A. Disease activity index (DAI) scoring criteria

| Percentage of Body Weight Loss | Stool Consistency | Hematochezia Grade | Score |
| --- | --- | --- | --- |
| 1-5 | Soft | Occult blood positivity | 1 |
| 5-10 | Unformed | Partial stool with gross blood | 2 |
| 10-20 | Diarrhea | Entire stool with gross blood | 3 |
| ≥20 | - | - | 4 |

Table B. Histopathological scoring criteria

| Dimension | Score | Criteria |
| --- | --- | --- |
| A. Inflammatory Cell Infiltration | 0 | None |
|  | 1 | Mild, limited to submucosa |
|  | 2 | Moderate, extending into the muscular layer |
|  | 3 | Severe, transmural (through all layers) |
| B. Architectural Alteration | 0 | Normal crypt structure |
|  | 1 | Destruction of the basal one-third of crypts |
|  | 2 | Destruction of the basal two-thirds of crypts |
|  | 3 | Only surface epithelium intact; severe crypt damage |
|  | 4 | Complete epithelial destruction with ulceration/necrosis |
| C. Mucosal Damage Depth | 0 | No damage |
|  | 1 | Mucosal layer |
|  | 2 | Submucosal layer |
|  | 3 | Muscular layer |
|  | 4 | Serosal layer (transmural) |
| D. Edema | 0 | None |
|  | 1 | Mild |
|  | 2 | Severe |

Table C. Primer sequences used for quantitative real-time PCR

|  | Forward Primer | Reverse Primer |
| --- | --- | --- |
| *Occludin* | CTGGATCTATGTACGGCTCACA | TCCACGTAGAGACCAGTACCT |
| *Claudin-1* | GGGGACAACATCGTGACCG | AGGAGTCGAAGACTTTGCACT |
| *iKB-α* | ACCTGGTTTCGCTCTTGTTGAAA | GCCTCCAAACACACAGTCATCAT |
| *CXCL 12* | CAAGTGTGCATTGACCCGAAATT | GAAGAGGGAGGAGCGAGTTACAA |
| *Cxcl13* | GGTGTTCTGGAGTGATTTCAACTG | ATTTGGCACGAGGATTCACACAT |
| *IL1β* | CAGCACATCAACAAGAGCTTCAG | GAGGATGGGCTCTTCTTCAAAGA |
| *TNF-α* | GCCTCCCTCTCATCAGTTCTATG | ACCTGGGAGTAGACAAGGTACAA |
| *Il-10* | GCTCTTACTGACTGGCATGAG | CGCAGCTCTAGGAGCATGTG |
| *Gapdh* | GGCATTGTGGAAGGGCTCAT | AGATCCACGACGGACACATT |


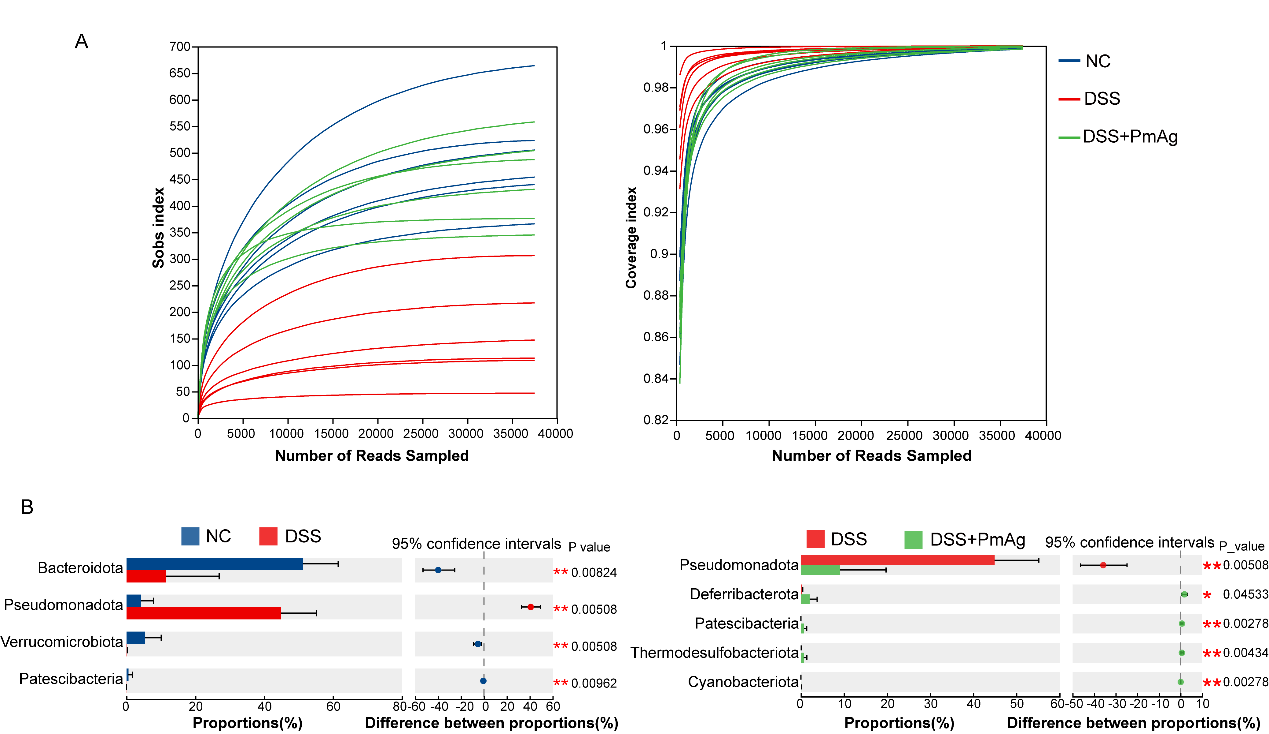


**Figure A. Intestinal microbiota diversity and phylum-level compositional changes in DSS-induced colitis following PmAg intervention**

(A) Rarefaction analysis of intestinal microbial communities. The Sobs index (left) reflects observed species richness across the Normal Control (NC), Dextran sulfate sodium–induced colitis (DSS), and DSS+PmAg groups, while the Coverage index (right) indicates sequencing depth sufficiency. Plateauing curves suggest adequate sequencing depth for capturing microbial diversity.

(B) Phylum-level relative abundance of intestinal microbiota and intergroup comparisons. The left panel shows the relative proportions of dominant bacterial phyla in the NC and DSS groups, and the right panel shows corresponding comparisons between the DSS and DSS+PmAg groups. Data are presented with 95% confidence intervals. Statistical significance was determined using the Wilcoxon rank-sum test (*P* < 0.05, **P* < 0.01).


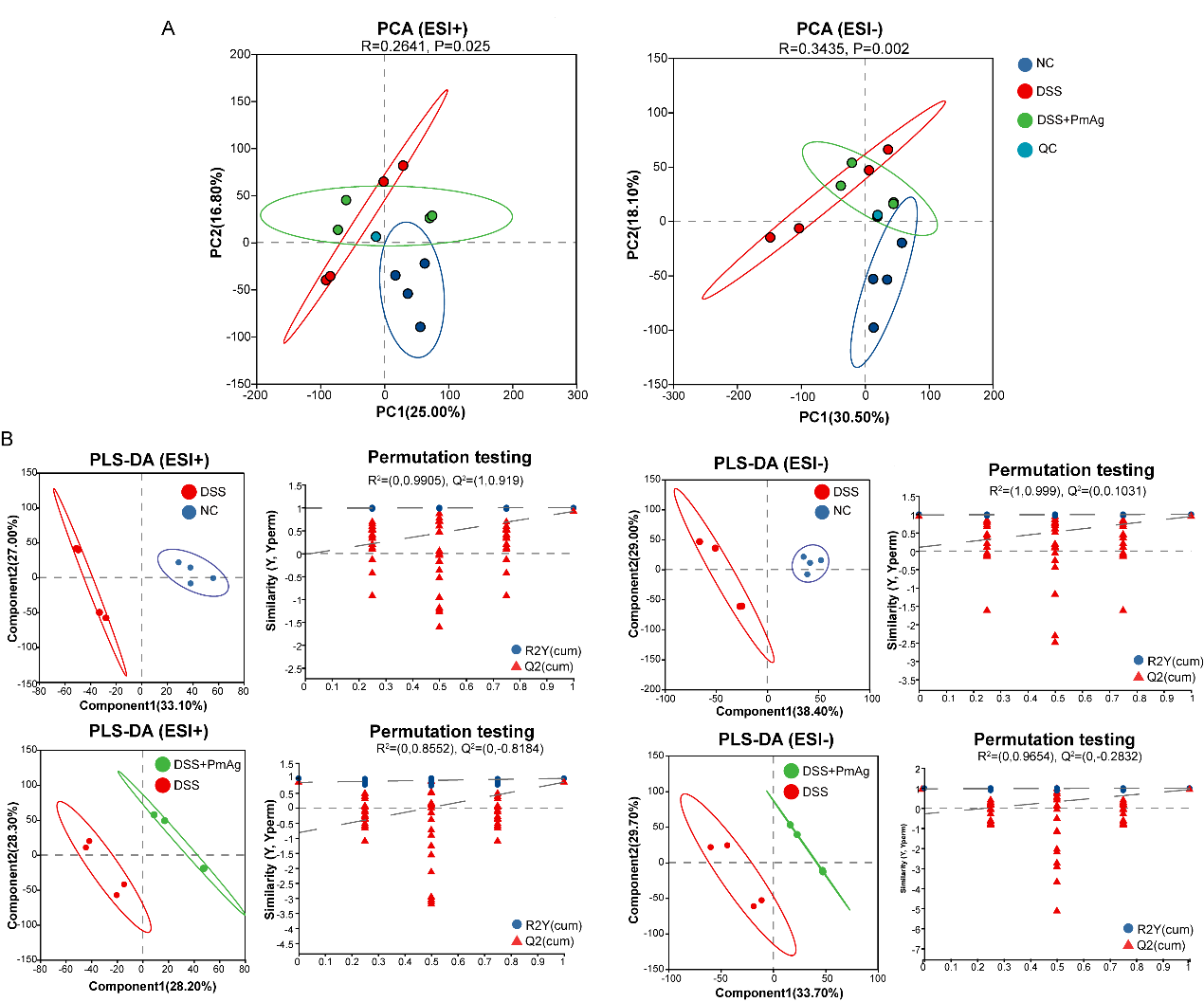


**Figure B. Multivariate statistical analysis of metabolomic profiles in DSS-induced colitis following PmAg intervention**

(A) Principal component analysis (PCA) of fecal metabolomic profiles acquired in positive (ESI+) and negative (ESI−) electrospray ionization modes. Score plots show the distribution of metabolic features among the Normal Control (NC), Dextran sulfate sodium–induced colitis (DSS), DSS+PmAg, and quality control (QC) groups. R and *P* values indicate overall group separation (ESI+: R = 0.2641, *P* = 0.025; ESI−: R = 0.3435, *P* = 0.002). Tight clustering of QC samples reflects analytical reproducibility.

(B) Partial least squares–discriminant analysis (PLS-DA) and corresponding permutation tests. The upper panels present PLS-DA models comparing the NC and DSS groups, while the lower panels show comparisons between the DSS and DSS+PmAg groups, under both ESI+ and ESI− modes. Permutation testing was used to assess model robustness, with R²Y indicating goodness of fit and Q²Y indicating predictive ability; all models exhibited Q²Y values greater than zero.
